# Supplementary material for: Small-scale distribution of microbes and biogeochemistry in the Great Barrier Reef
Source: PeerJ. 2020 Oct 21;8:e10049. doi: 10.7717/peerj.10049 (PMC7585385; doi:10.7717/peerj.10049)
Supplement: Supplemental Information 1 — R2 and p values between each parameter measured (nitrate/nitrite - NO3−/NO2−; phosphate - HPO42−; dissolved organic carbon –DOC; total dissolved nitrogen - TDN; chlorophylla - chla; and bacterial and viral abundances) during the spatial study for all sites together, and individually in the Great Barrier Reef; n/a. –not applicable. Please note in bold the statistically significant correlations. [file peerj-08-10049-s001.docx]

|  |  | **R^2^/p-value** | | | | | | |
| --- | --- | --- | --- | --- | --- | --- | --- | --- |
| **Sites** | **Parameters** | **NO_3_^-^/NO_2_^-^** | **HPO_4_^2-^** | **DOC** | **TDN** | **Chl *a*** | **Bacteria** |  |
| **All sites** | **NO_3_^-^/NO_2_^-^** | - |  |  |  |  |  |  |
|  | **HPO_4_^2-^** | 0.28/0.**001** | - |  |  |  |  |  |
|  | **DOC** | 0.18/**0.023** | 0.24/**0.003** | - |  |  |  |  |
|  | **TDN** | 0.04/0.334 | -0.05/0.307 | 0.16/**0.040** | - |  |  |  |
|  | **Chl *a*** | 0.20/**0.014** | -0.07/0.223 | -0.04/0.340 | -0.11/0.105 | - |  |  |
|  | **Bacteria** | 0.07/0.205 | -0.54/**0.000** | -0.22/**0.007** | -0.04/0.327 | 0.18/**0.024** | - |  |
|  | **Viruses** | 0.03/0.367 | -0.58/**0.000** | -0.18/**0.023** | 0.17/**0.027** | 0.27/**0.001** | 0.55/**0.000** |  |
| **Site 1** | **NO_3_^-^/NO_2_^-^** | - |  |  |  |  |  |  |
|  | **HPO_4_^2-^** | 0.32/0.063 | - |  |  |  |  |  |
|  | **DOC** | 0.37/**0.036** | -0.33/0.054 | - |  |  |  |  |
|  | **TDN** | 0.31/0.068 | 0.28/0.091 | 0.10/0.311 | - |  |  |  |
|  | **Chl *a*** | 0.28/0.089 | -0.15/0.243 | 0.00/0.500 | 0.16/0.219 | - |  |  |
|  | **Bacteria** | -0.29/0.080 | -0.17/0.211 | -0.01/0.475 | -0.08/0.353 | 0.01/0.322 | - |  |
|  | **Viruses** | -0.14/0.257 | -0.48/**0.008** | -0.01/0.477 | -0.25/0.115 | 0.15/0.241 | -0.13/0.268 |  |
| **Site 2** | **NO_3_^-^/NO_2_^-^** | - |  |  |  |  |  |  |
|  | **HPO_4_^2-^** | n/a | - |  |  |  |  |  |
|  | **DOC** | 0.05/0.400 | n/a | - |  |  |  |  |
|  | **TDN** | 0.48/**0.008** | n/a | -0.06/0.385 | - |  |  |  |
|  | **Chl *a*** | -0.43/**0.016** | n/a | -0.05/0.412 | -0.26/0.107 | - |  |  |
|  | **Bacteria** | -0.12/0.288 | n/a | -0.10/0.312 | -0.24/0.121 | 0.20/0.167 | - |  |
|  | **Viruses** | 0.43/**0.016** | n/a | 0.00/0.494 | 0.08/0.362 | -0.09/0.329 | -0.33/0.056 |  |
| **Site 3** | **NO_3_^-^/NO_2_^-^** | - |  |  |  |  |  |  |
|  | **HPO_4_^2-^** | 0.31/0.067 | - |  |  |  |  |  |
|  | **DOC** | -0.02/0.464 | 0.37/**0.034** | - |  |  |  |  |
|  | **TDN** | 0.12/0.277 | 0.13/0.263 | 0.28/0.085 | - |  |  |  |
|  | **Chl *a*** | 0.02/0.467 | 0.01/0.490 | -0.04/0.419 | 0.09/0.334 | - |  |  |
|  | **Bacteria** | -0.27/0.094 | 0.16/0.228 | -0.17/0.211 | -0.35/**0.044** | 0.06/0.383 | - |  |
|  | **Viruses** | 0.07/0.365 | 0.12/0.282 | -0.20/0.173 | 0.10/0.310 | 0.33/0.054 | 0.50/**0.006** |  |
| **Site 4** | **NO_3_^-^/NO_2_^-^** | - |  |  |  |  |  |  |
|  | **HPO** | 0.47/**0.009** | - |  |  |  |  |  |
|  | **DOC** | 0.39/**0.026** | 0.43/**0.017** | - |  |  |  |  |
|  | **TDN** | -0.13/0.264 | -0.07/0.373 | -0.37/**0.035** | - |  |  |  |
|  | **Chl *a*** | 0.32/0.058 | 0.28/0.090 | 0.20/0.174 | -0.39/**0.028** | - |  |  |
|  | **Bacteria** | -0.04/0.433 | -0.10/0.315 | -0.31/0.066 | -0.07/0.364 | 0.06/0.389 | - |  |
|  | **Viruses** | -0.07/0.364 | -0.26/0.103 | 0.07/0.371 | 0.07/0.037 | 0.00/0.495 | -0.32/0.057 |  |
| **Site 5** | **NO_3_^-^/NO_2_^-^** | - |  |  |  |  |  |  |
|  | **HPO_4_^2-^** | 0.52/**0.004** | - |  |  |  |  |  |
|  | **DOC** | 0.11/0.307 | 0.01/0.489 | - |  |  |  |  |
|  | **TDN** | 0.23/0.135 | 0.00/0.497 | 0.06/0.388 | - |  |  |  |
|  | **Chl *a*** | -0.12/0.290 | 0.01/0.485 | 0.09/0.329 | 0.00/0.498 | - |  |  |
|  | **Bacteria** | 0.07/0.374 | -0.04/0.429 | 0.19/0.185 | 0.09/0.335 | 0.07/0.367 | - |  |
|  | **Viruses** | 0.23/0.130 | 0.07/0.368 | -0.17/0.208 | 0.30/0.074 | -0.42/**0.020** | -0.08/0.355 |  |
