# Supplementary material for: Small-scale distribution of microbes and biogeochemistry in the Great Barrier Reef
Source: PeerJ. 2020 Oct 21;8:e10049. doi: 10.7717/peerj.10049 (PMC7585385; doi:10.7717/peerj.10049)
Supplement: Supplemental Information 2 — R2 and p values between each parameter measured (nitrate/nitrite - NO3−/NO2−; phosphate - HPO42−; dissolved organic carbon –DOC; total dissolved nitrogen - TDN; chlorophyll a - chl a; and bacterial and viral abundances) during the spatial study for all sites together, and individually in the Great Barrier Reef. TDN correlations are not shown for days 2 and 3 due to missing values; n/a. –not applicable. Please note in bold the statistically significant correlations. [file peerj-08-10049-s002.docx]

|  |  | **R^2^/p-value** | | | | | |
| --- | --- | --- | --- | --- | --- | --- | --- |
| **Days** | **Parameters** | **NO_3_^-^/NO_2_^-^** | **HPO_4_^2-^** | **DOC** | **TDN** | **Chl *a*** | **Bacteria** |
| **All days** | **NO_3_^-^/NO_2_^-^** | - |  |  |  |  |  |
|  | **HPO_4_^2-^** | 0.63/**0.000** | - |  |  |  |  |
|  | **DOC** | -0.28/**0.004** | -0.02/0.473 | - |  |  |  |
|  | **Chl *a*** | -0.11/0.117 | -0.25/**0.004** | -0.23/**0.008** | n/a | - |  |
|  | **Bacteria** | -0.75/**0.000** | -0.50/**0.000** | 0.00/0.445 | n/a | 0.10/0.151 | - |
|  | **Viruses** | -0.54/**0.000** | -0.29/**0.002** | 0.14/0.093 | n/a | -0.10/0.170 | 0.38/**0.000** |
| **Day 1** | **NO_3_^-^/NO_2_^-^** | - |  |  |  |  |  |
|  | **HPO_4_^2-^** | 0.15/0.236 | - |  |  |  |  |
|  | **DOC** | -0.05/0.402 | 0.32/0.060 | - |  |  |  |
|  | **TDN** | -0.03/0.447 | 0.03/0.455 | 0.57/**0.002** | - |  |  |
|  | **Chl *a*** | -0.20/0.155 | 0.03/0.086 | -0.31/0.057 | -0.19/0.239 | - |  |
|  | **Bacteria** | -0.03/0.445 | 0.17/0.202 | -0.07/0.376 | 0.09/0.343 | -0.26/0.112 | - |
|  | **Viruses** | 0.18/0.188 | 0.26/0.103 | 0.17/0.212 | 0.09/0.344 | -0.36/**0.046** | 0.06/0.379 |
| **Day 2** | **NO_3_^-^/NO_2_^-^** | - |  |  |  |  |  |
|  | **HPO_4_^2-^** | 0.35/**0.045** | - |  |  |  |  |
|  | **DOC** | 0.14/0.249 | 0.28/0.092 | - |  |  |  |
|  | **Chl *a*** | 0.02/0.460 | -0.24/0.126 | -0.24/0.127 | n/a | - |  |
|  | **Bacteria** | -0.18/0.199 | -0.18/0.200 | -0.23/0.138 | n/a | -0.04/0.429 | - |
|  | **Viruses** | 0.35/0.045 | 0.35/**0.044** | 0.08/0.358 | n/a | -0.01/0.479 | 0.10/0.311 |
| **Day 3** | **NO_3_^-^/NO_2_^-^** | - |  |  |  |  |  |
|  | **HPO_4_^2-^** | 0.16/0.223 | - |  |  |  |  |
|  | **DOC** | 0.42/**0.019** | 0.22/0.141 | - |  |  |  |
|  | **Chl *a*** | 0.39/**0.027** | -0.12/0.280 | -0.18/0.195 | n/a | - |  |
|  | **Bacteria** | -0.53/**0.003** | 0.05/0.402 | -0.56/**0.002** | n/a | 0.03/0.442 | - |
|  | **Viruses** | -0.15/0.244 | 0.21/0.157 | -0.14/0.248 | n/a | -0.11/0.296 | -0.03/0.440 |
| **Day 4** | **NO_3_^-^/NO_2_^-^** | - |  |  |  |  |  |
|  | **HPO_4_^2-^** | -0.04/0.431 | - |  |  |  |  |
|  | **DOC** | -0.19/0.392 | -0.20/0.178 | - |  |  |  |
|  | **TDN** | 0.099/0.319 | -0.433/**0.015** | 0.43/**0.019** | - |  |  |
|  | **Chl *a*** | -0.03/0.437 | -0.32/0.062 | -0.10/0.315 | 0.213/0.153 | - |  |
|  | **Bacteria** | -0.44/**0.014** | -0.04/0.421 | 0.15/0.320 | 0.18/0.189 | 0.02/0.470 | - |
|  | **Viruses** | -0.65/**0.000** | -0.03/0.450 | 0.14/0.336 | 0.02/0.467 | -0.16/0.227 | 0.34/**0.047** |
