# Supplementary material for: Small-scale distribution of microbes and biogeochemistry in the Great Barrier Reef
Source: PeerJ. 2020 Oct 21;8:e10049. doi: 10.7717/peerj.10049 (PMC7585385; doi:10.7717/peerj.10049)
Supplement: Supplemental Information 3 — Spearman correlation coefficient (Rs) and p values between each parameter measured (nitrate/nitrite - NO3−/NO2−; phosphate - HPO42−; dissolved organic carbon –DOC; total dissolved nitrogen - TDN; chlorophyll a - chl a; and bacterial and viral abundances) during the spatial study for all sites together, and individually in the Great Barrier Reef; n/a. –not applicable. Please note in bold the statistically significant correlations. [file peerj-08-10049-s003.docx]

|  |  | R_s_**/p-value** | | | | | | |
| --- | --- | --- | --- | --- | --- | --- | --- | --- |
| **Sites** | **Parameters** | **NO_3_^-^/NO_2_^-^** | **HPO_4_^2-^** | **DOC** | **TDN** | **Chl *a*** | **Bacteria** |  |
| **All sites** | **NO_3_^-^/NO_2_^-^** | - |  |  |  |  |  |  |
|  | **HPO_4_^2-^** | 0.33/0.**000** | - |  |  |  |  |  |
|  | **DOC** | 0.06/0.502 | 0.259/**0.004** | - |  |  |  |  |
|  | **TDN** | -0.01/0.943 | 0.00/0.998 | 0.17/0.057 | - |  |  |  |
|  | **Chl *a*** | 0.12/0.195 | 0.069/0.445 | -0.06/0.340 | -0.16/0.071 | - |  |  |
|  | **Bacteria** | 0.11/0.236 | -0.54/**0.000** | -0.26/**0.004** | 0.05/0.596 | 0.11/0.232 | - |  |
|  | **Viruses** | 0.02/0.796 | -0.46/**0.000** | -0.22/0.012 | 0.12/0.203 | 0.29/**0.001** | 0.27/**0.003** |  |
| **Site 1** | **NO_3_^-^/NO_2_^-^** | - |  |  |  |  |  |  |
|  | **HPO_4_^2-^** | 0.63/**0.001** | - |  |  |  |  |  |
|  | **DOC** | 0.07/0.728 | -0.24/0.244 | - |  |  |  |  |
|  | **TDN** | 0.49/**0.012** | 0.39/0.057 | 0.18/0.388 | - |  |  |  |
|  | **Chl *a*** | 0.12/0.577 | -0.16/0.435 | -0.14/0.492 | 0.17/0.425 | - |  |  |
|  | **Bacteria** | -0.32/0.123 | -0.11/0.598 | 0.04/0.834 | -0.17/0.405 | -0.26/0.211 | - |  |
|  | **Viruses** | -0.33/0.105 | -0.47/**0.017** | -0.03/0.889 | -0.28/0.169 | 0.17/0.414 | -0.19/0.363 |  |
| **Site 2** | **NO_3_^-^/NO_2_^-^** | - |  |  |  |  |  |  |
|  | **HPO_4_^2-^** | n/a | - |  |  |  |  |  |
|  | **DOC** | 0.10/0.624 | n/a | - |  |  |  |  |
|  | **TDN** | 0.45/**0.024** | n/a | 0.24/0.241 | - |  |  |  |
|  | **Chl *a*** | -0.39/0.058 | n/a | 0.06/0.765 | -0.21/0.304 | - |  |  |
|  | **Bacteria** | -0.14/0.514 | n/a | 0.03/0.894 | -0.11/0.613 | 0.26/0.217 | - |  |
|  | **Viruses** | 0.41/**0.041** | n/a | -0.27/0.197 | 0.09/0.683 | -0.14/0.512 | -0.31/**0.013** |  |
| **Site 3** | **NO_3_^-^/NO_2_^-^** | - |  |  |  |  |  |  |
|  | **HPO_4_^2-^** | 0.31/0.137 | - |  |  |  |  |  |
|  | **DOC** | -0.09/0.645 | 0.27/0.189 | - |  |  |  |  |
|  | **TDN** | 0.32/0.123 | 0.18/0.379 | 0.19/0.357 | - |  |  |  |
|  | **Chl *a*** | 0.08/0.712 | 0.05/0.820 | -0.01/0.956 | -0.11/0.604 | - |  |  |
|  | **Bacteria** | -0.41/**0.041** | -0.30/0.892 | -0.05/0.802 | -0.42/**0.034** | 0.04/0.861 | - |  |
|  | **Viruses** | 0.12/0.548 | 0.09/0.651 | -0.20/0.173 | 0.18/0.378 | 0.28/0.169 | -0.12/0.581 |  |
| **Site 4** | **NO_3_^-^/NO_2_^-^** | - |  |  |  |  |  |  |
|  | **HPO** | 0.40/**0.045** | - |  |  |  |  |  |
|  | **DOC** | 0.31/0.133 | 0.22/0.286 | - |  |  |  |  |
|  | **TDN** | -0.22/0.280 | 0.00/0.992 | -0.28/0.181 | - |  |  |  |
|  | **Chl *a*** | 0.19/0.365 | 0.22/0.295 | 0.14/0.502 | -0.32/0.121 | - |  |  |
|  | **Bacteria** | 0.09/0.672 | -0.02/0.926 | -0.34/0.099 | -0.05/0.812 | 0.00/0.999 | - |  |
|  | **Viruses** | -0.33/0.102 | -0.33/0.106 | 0.15/0.474 | 0.03/0.904 | -0.02/0.919 | -0.45/**0.022** |  |
| **Site 5** | **NO_3_^-^/NO_2_^-^** | - |  |  |  |  |  |  |
|  | **HPO_4_^2-^** | 0.17/0.421 | - |  |  |  |  |  |
|  | **DOC** | 0.11/0.599 | 0.06/0.764 | - |  |  |  |  |
|  | **TDN** | 0.30/0.146 | 0.03/0.900 | 0.19/0.347 | - |  |  |  |
|  | **Chl *a*** | -0.14/0.500 | -0.04/0.832 | 0.06/0.760 | 0.03/0.899 | - |  |  |
|  | **Bacteria** | 0.29/0.162 | 0.02/0.945 | 0.11/0.616 | 0.22/0.285 | 0.10/0.621 | - |  |
|  | **Viruses** | 0.26/0.205 | -0.00/0.991 | -0.03/0.907 | 0.19/0.366 | -0.54/**0.006** | 0.06/0.777 |  |
