# Supplementary material for: Small-scale distribution of microbes and biogeochemistry in the Great Barrier Reef
Source: PeerJ. 2020 Oct 21;8:e10049. doi: 10.7717/peerj.10049 (PMC7585385; doi:10.7717/peerj.10049)
Supplement: Supplemental Information 4 — Spearman correlation coefficient (Rs) and p values between each parameter measured (nitrate/nitrite - NO3−/NO2−; phosphate - HPO42−; dissolved organic carbon –DOC; total dissolved nitrogen - TDN; chlorophyll a - chl a; and bacterial and viral abundances) during the temporal study for all sites together, and individually in the Great Barrier Reef. TDN correlations are not shown for days 2 and 3 due to missing values; n/a. –not applicable. Please note in bold the statistically significant correlations. [file peerj-08-10049-s004.docx]

|  |  | R_s_**/p-value** | | | | | |
| --- | --- | --- | --- | --- | --- | --- | --- |
| **Days** | **Parameters** | **NO_3_^-^/NO_2_^-^** | **HPO_4_^2-^** | **DOC** | **TDN** | **Chl *a*** | **Bacteria** |
| **All days** | **NO_3_^-^/NO_2_^-^** | - |  |  |  |  |  |
|  | **HPO_4_^2-^** | 0.65/**0.000** | - |  |  |  |  |
|  | **DOC** | -0.12/0.256 | 0.12/0.234 | - |  |  |  |
|  | **Chl *a*** | -0.17/0.085 | -0.24/**0.015** | -0.24/**0.015** | n/a | - |  |
|  | **Bacteria** | -0.72/**0.000** | -0.47/**0.000** | 0.16/0.107 | n/a | 0.06/0.539 | - |
|  | **Viruses** | -0.29/**0.003** | -0.26/**0.008** | 0.17/0.092 | n/a | -0.16/0.101 | 0.35/**0.000** |
| **Day 1** | **NO_3_^-^/NO_2_^-^** | - |  |  |  |  |  |
|  | **HPO_4_^2-^** | 0.23/0.258 | - |  |  |  |  |
|  | **DOC** | 0.06/0.776 | 0.24/0.249 | - |  |  |  |
|  | **TDN** | 0.18/0.382 | 0.05/0.814 | 0.52/**0.008** | - |  |  |
|  | **Chl *a*** | -0.37/0.071 | -0.13/0.534 | -0.19/0.348 | -0.26/0.209 | - |  |
|  | **Bacteria** | 0.20/0.331 | 0.16/0.432 | -0.17/0.404 | 0.12/0.564 | -0.18/0.38 | - |
|  | **Viruses** | 0.02/0.944 | 0.28/0.171 | 0.27/0.278 | 0.22/0.303 | -0.37/0.067 | 0.14/0.493 |
| **Day 2** | **NO_3_^-^/NO_2_^-^** | - |  |  |  |  |  |
|  | **HPO_4_^2-^** | 0.36/0.078 | - |  |  |  |  |
|  | **DOC** | 0.19/0.369 | 0.43/**0.031** | - |  |  |  |
|  | **Chl *a*** | 0.06/0.764 | -0.12/0.581 | -0.14/0.506 | n/a | - |  |
|  | **Bacteria** | -0.06/0.761 | -0.23/0.273 | -0.17/0.425 | n/a | -0.00/0.995 | - |
|  | **Viruses** | 0.36/0.081 | 0.38/0.064 | 0.16/0.455 | n/a | -0.17/0.408 | 0.06/0.764 |
| **Day 3** | **NO_3_^-^/NO_2_^-^** | - |  |  |  |  |  |
|  | **HPO_4_^2-^** | 0.17/0.424 | - |  |  |  |  |
|  | **DOC** | 0.35/0.081 | 0.18/0.389 | - |  |  |  |
|  | **Chl *a*** | 0.49/**0.012** | -0.28/0.181 | -0.15/0.471 | n/a | - |  |
|  | **Bacteria** | -0.51/**0.009** | 0.08/0.713 | -0.55/**0.004** | n/a | -0.16/0.442 | - |
|  | **Viruses** | -0.20/0.338 | 0.33/0.106 | -0.06/0.767 | n/a | -0.17/0.407 | -0.07/0.728 |
| **Day 4** | **NO_3_^-^/NO_2_^-^** | - |  |  |  |  |  |
|  | **HPO_4_^2-^** | -0.05/0.829 | - |  |  |  |  |
|  | **DOC** | -0.13/0.545 | -0.35/0.084 | - |  |  |  |
|  | **TDN** | 0.14/0.492 | -0.39/**0.053** | 0.48/**0.015** | - |  |  |
|  | **Chl *a*** | -0.12/0.573 | -0.29/0.166 | -0.12/0.564 | 0.26/0.211 | - |  |
|  | **Bacteria** | -0.52/**0.008** | -0.09/0.658 | 0.17/0.426 | 0.11/0.612 | 0.06/0.768 | - |
|  | **Viruses** | -0.63/**0.001** | -0.07/0.725 | 0.07/0.732 | 0.00/0.994 | -0.04/0.855 | 0.36/0.079 |
