# Supplementary material for: Small-scale distribution of microbes and biogeochemistry in the Great Barrier Reef
Source: PeerJ. 2020 Oct 21;8:e10049. doi: 10.7717/peerj.10049 (PMC7585385; doi:10.7717/peerj.10049)
Supplement: Supplemental Information 5 — p values of these tests for multiple comparisons of independent samples (with Bonferroni adjustment) for each parameter measured (nitrate/nitrite - NO3−/NO2−; phosphate - HPO42−; dissolved organic carbon –DOC; total dissolved nitrogen - TDN; chlorophyll a - chl a; and bacterial and viral abundances) at sites 1 to 5, included in the spatial study in the Great Barrier Reef. The pairs with significant differences are pointed out. [file peerj-08-10049-s005.docx]

|  |  | **Dunn's test (Bonferroni adjustment)** | |
| --- | --- | --- | --- |
| **Parameters** | **p values of Kruskal-Wallis test** | **p values** | **True differences between sites** |
| **NO_3_^-^/NO_2_^-^** | 1.9 x10^-6^ | 4.5 x 10^-3^ | 1-3 |
|  |  | 7.3 x 10^-5^ | 2-3 |
|  |  | 5.5 x 10^-4^ | 3-5 |
| **HPO_4_^2-^** | 4.5 x 10^-14^ | 4.4 x 10^-4^ | 1-2 |
|  |  | 1.7 x 10^-12^ | 2-4 |
|  |  | 3.5 x 10^-6^ | 3-4 |
|  |  | 8.3 x 10^-10^ | 4-5 |
| **DOC** | 3.0 x 10^-4^ | 1.2 x 10^-3^ | 1-2 |
|  |  | 3.7 x 10^-3^ | 2-4 |
| **TDN** | 2.4 x 10^-11^ | 3.3 x 10^-7^ | 1-2 |
|  |  | 3.1 x 10^-4^ | 1-3 |
|  |  | 2.1 x 10^-7^ | 1-4 |
|  |  | 3.1 x 10^-5^ | 2-5 |
|  |  | 0.010 | 3-5 |
|  |  | 2.2 x 10^-5^ | 4-5 |
| **Chl *a*** | 5.3 x 10^-5^ | 4.0 x 10^-4^ | 2-5 |
|  |  | 6.8 x 10^-4^ | 3-5 |
| **Bacteria** | < 2.2 x 10^-16^ | 7.4 x 10^-12^ | 1-3 |
|  |  | 7.5 x 10^-5^ | 1-5 |
|  |  | 8.1 x 10^-5^ | 2-3 |
|  |  | 1.8 x 10^-6^ | 2-4 |
|  |  | <2.0 x 10^-16^ | 3-4 |
|  |  | 2.8 x 10^-11^ | 4-5 |
| **Viruses** | < 2.2 x 10^-16^ | 1.1 x 10^-11^ | 1-4 |
|  |  | 1.0 x 10^-3^ | 1-5 |
|  |  | < 2 x 10^-16^ | 2-4 |
|  |  | 3.8 x 10^-7^ | 3-5 |
|  |  | 1.2 x 10^-6^ | 3-4 |
