# Supplementary material for: Small-scale distribution of microbes and biogeochemistry in the Great Barrier Reef
Source: PeerJ. 2020 Oct 21;8:e10049. doi: 10.7717/peerj.10049 (PMC7585385; doi:10.7717/peerj.10049)
Supplement: Supplemental Information 6 — p values of these tests for multiple comparisons of independent samples (with Bonferroni adjustment) for each parameter measured (nitrate/nitrite - NO3−/NO2−; phosphate - HPO42−; dissolved organic carbon –DOC; total dissolved nitrogen - TDN; chlorophyll a - chl a; and bacterial and viral abundances) at the 4 days of the temporal study at Bowling Green Bay (site 6) in the Great Barrier Reef. The pairs with significant differences are pointed out. [file peerj-08-10049-s006.docx]

|  |  | **Dunn's test (Bonferroni adjustment)** | |
| --- | --- | --- | --- |
| **Parameters** | **p values of Kruskal-Wallis test** | **p values** | **True differences between days** |
| **NO_3_^-^/NO_2_^-^** | 2.2 x10^-16^ | 0.068 | 1-2 |
|  |  | 4.7 x 10^-8^ | 1-3 |
|  |  | < 2.0 x 10^-16^ | 1-4 |
|  |  | 7.2 x 10^-3^ | 2-3 |
|  |  | 1.2 x 10^-9^ | 2-4 |
|  |  | 0.010 | 3-4 |
| **HPO_4_^2-^** | 2.1 x 10^-10^ | 3.1 x 10^-3^ | 1-3 |
|  |  | 4.5 x 10^-6^ | 1-4 |
|  |  | 3.8 x 10^-5^ | 2-3 |
|  |  | 1.3 x 10^-8^ | 2-4 |
| **DOC** | 1.0 x 10^-4^ | 2.5 x 10^-3^ | 1-2 |
|  |  | 4.1 x 10^-3^ | 1-3 |
|  |  | 1.2 x 10^-3^ | 1-4 |
|  |  | 2.7 x 10^-11^ | 2-3 |
|  |  | 6.5 10^-12^ | 3-4 |
| **Chl *a*** | 0.030 | 0.035 | 1-3 |
| **Bacteria** | 1.8 x 10^-11^ | 4.8 x 10^-11^ | 1-4 |
|  |  | 1.5 x 10^-7^ | 2-4 |
|  |  | 1.3 x 10^-4^ | 3-4 |
| **Viruses** | 6.5 x 10^-13^ | 5.8 x 10^-6^ | 1-2 |
|  |  | 0.010 | 1-3 |
|  |  | 7.5 x 10^-12^ | 2-4 |
|  |  | 8.7 x 10^-7^ | 3-4 |
