# Supplementary material for: Small-scale distribution of microbes and biogeochemistry in the Great Barrier Reef
Source: PeerJ. 2020 Oct 21;8:e10049. doi: 10.7717/peerj.10049 (PMC7585385; doi:10.7717/peerj.10049)
Supplement: Supplemental Information 7 — The samples were taken fornitrate/nitrite (NO3−/NO2−), phosphate (HPO42−), dissolved organic carbon (DOC), total dissolved nitrogen (TDN), chlorophyll a (chl a), bacterial and viral abundances, and virus to bacteria ratio (VBR) at days 1 to 4 at Bowling Green Bay (site 6) in the Great Barrier Reef (Australia); n.d. - not determined. [file peerj-08-10049-s007.docx]

| **Day** | **NO_3_^-^/NO_2_^-^ (µmol l^-1^)** | **HPO_4_^2-^ (µmol L^-1^)** | **DOC (µmol l^-1^)** | **TDN (µmol l^-1^)** | **Chl *a* (µg l^-1^)** | **Bacteria (x10^5^ ml^-1^)** | **Viruses (x10^5^ ml^-1^)** | **VBR** |
| --- | --- | --- | --- | --- | --- | --- | --- | --- |
| 1 | 0.05 | 0.42 | 121 | 10.4 | 0.04 | 19.8 | 111.2 | 5.6 |
| 2 | 0.08 | 0.08 | 103 | n.d. | 0.21 | 18.0 | 100.7 | 5.6 |
| 3 | 0.24 | 0.11 | 114 | 10.4 | 0.10 | 19.0 | 92.0 | 4.9 |
| 4 | 0.65 | 0.10 | 101 | 11.1 | 0.24 | 15.1 | 96.8 | 6.4 |
