# Supplementary figures and images for: Small-scale distribution of microbes and biogeochemistry in the Great Barrier Reef
Source: PeerJ. 2020 Oct 21;8:e10049. doi: 10.7717/peerj.10049 (PMC7585385; doi:10.7717/peerj.10049)

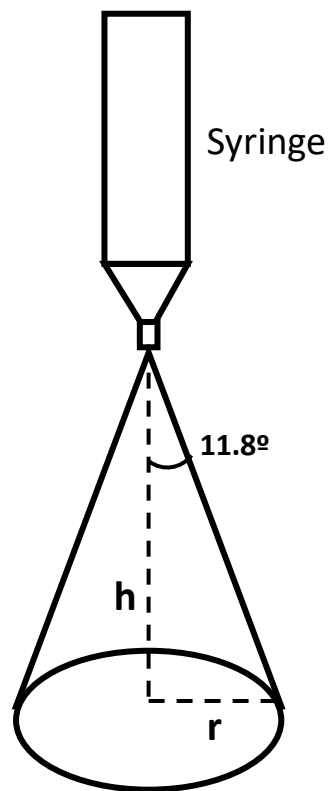

Supplement: Supplemental Information 8 — It isapplied to the rapid intake of water by the syringes in the water column. It is assumed that r is the minimum distance between the syringes without interfering with neighbouring sampling volumes; h is the height of the cone and the tan θ is 11.8 (Pope, 2000). [file peerj-08-10049-s008.pdf]

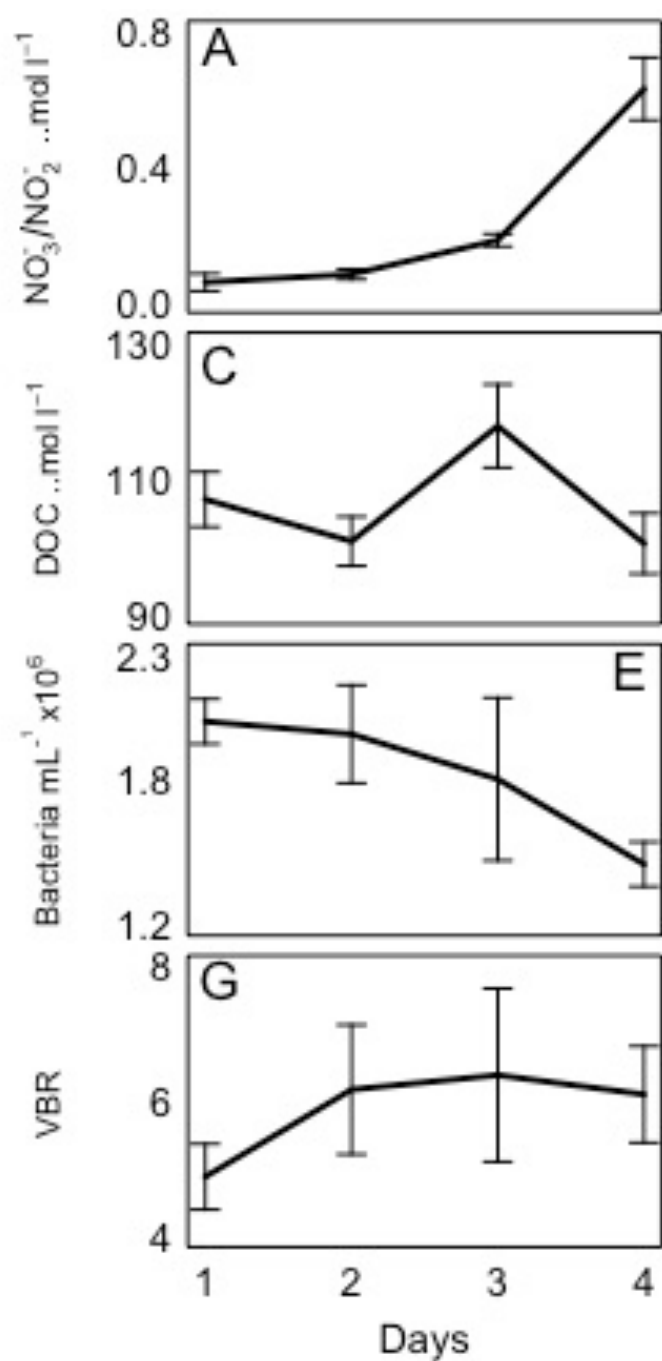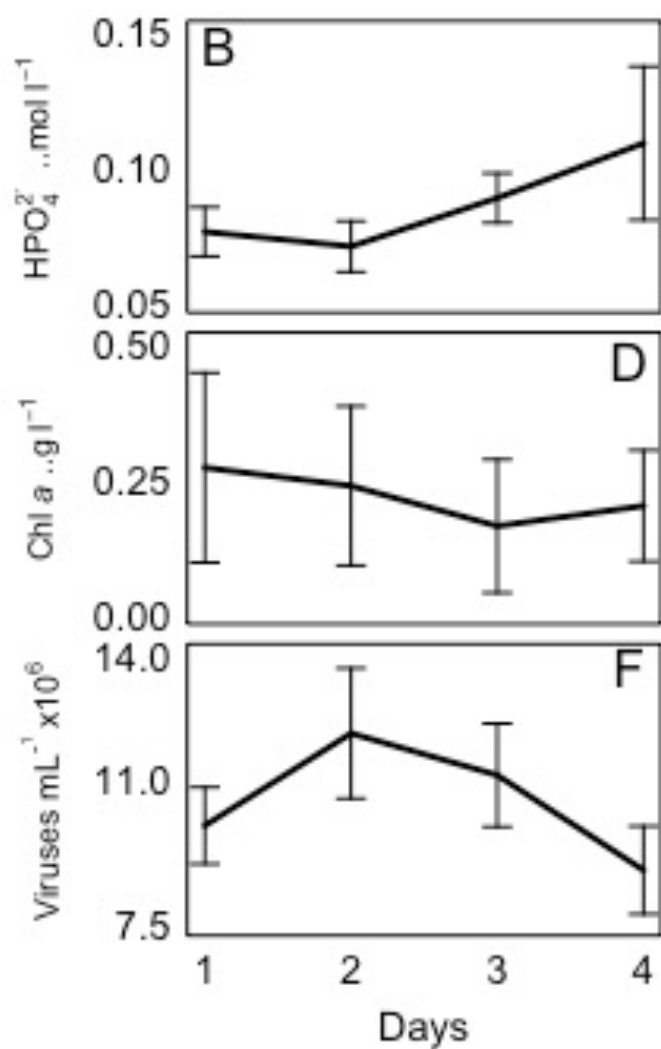

Supplement: Supplemental Information 9 — Line graphics showing average values (n = 25) for each of the 4 days for all the parameters measured in the Great Barrier Reef (Australia): (A) nitrate/nitrite - NO3−/NO2−, (B) phosphate - HPO42−, (C) dissolved organic carbon - DOC, (D) chlorophyll a - chl a, (E) bacteria, (F) viruses and (G) virus to microbe ratio VBR, excluding TDN has there were few values at days 2 and 3. Bars represent standard deviations. [file peerj-08-10049-s009.pdf]

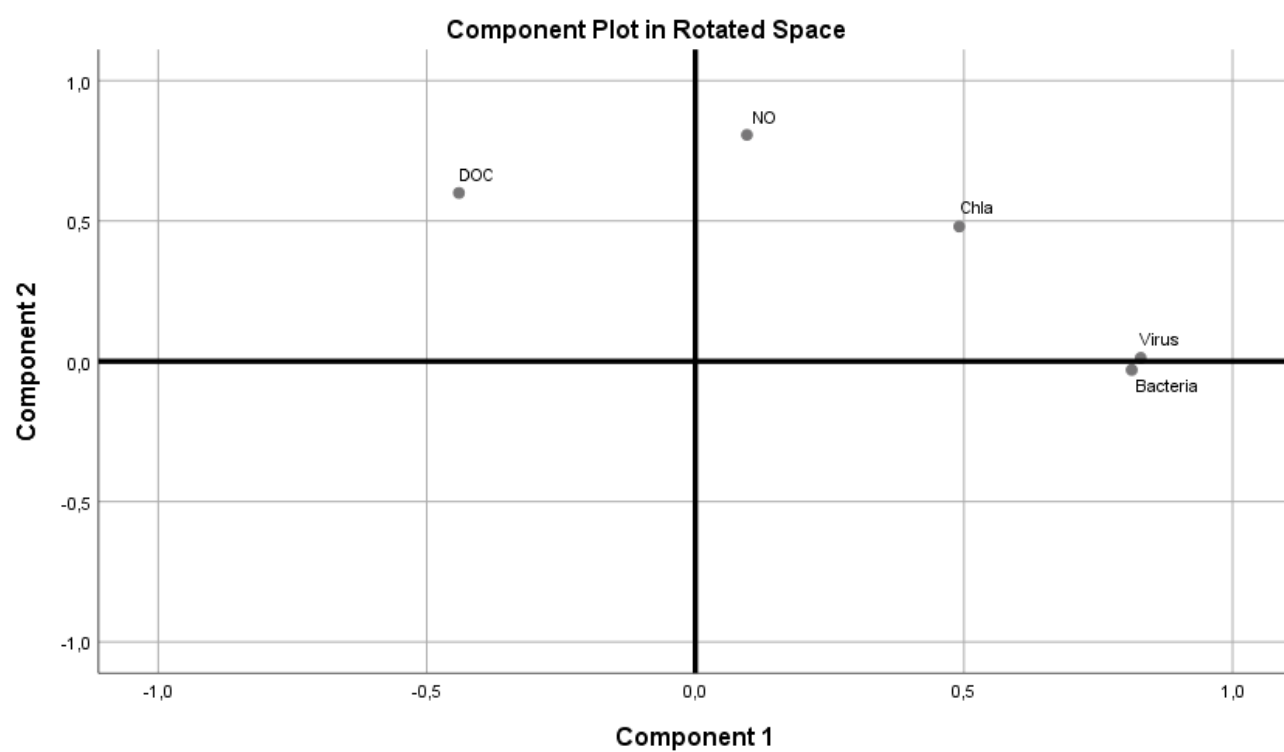

Supplement: Supplemental Information 10 — From the spatial data (sites 1, 2, 3, 4, and 5) collected in the Great Barrier Reef (Australia) displaying the two factors (chemical: NO3−/NO2− and DOC; and biological: bacteria, chl a and viruses) using correlation matrix, excluding HPO42−and TDN data. Please note that NO regards NO3−/NO2−. [file peerj-08-10049-s010.pdf]

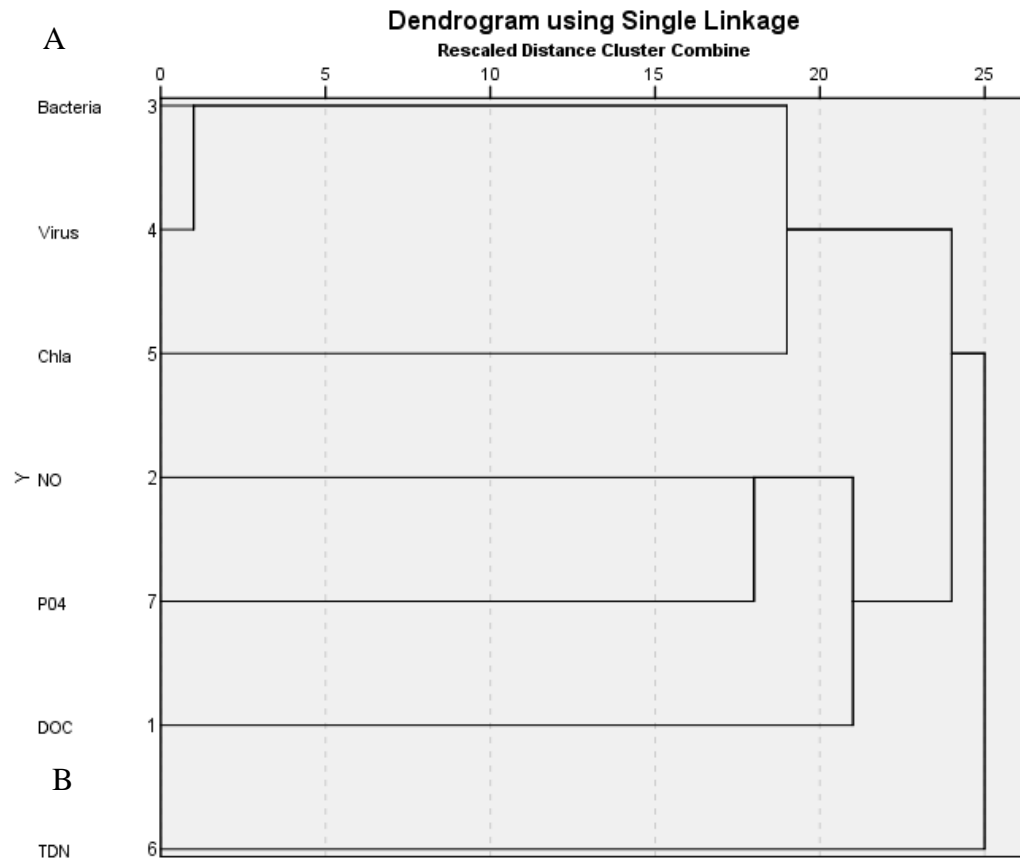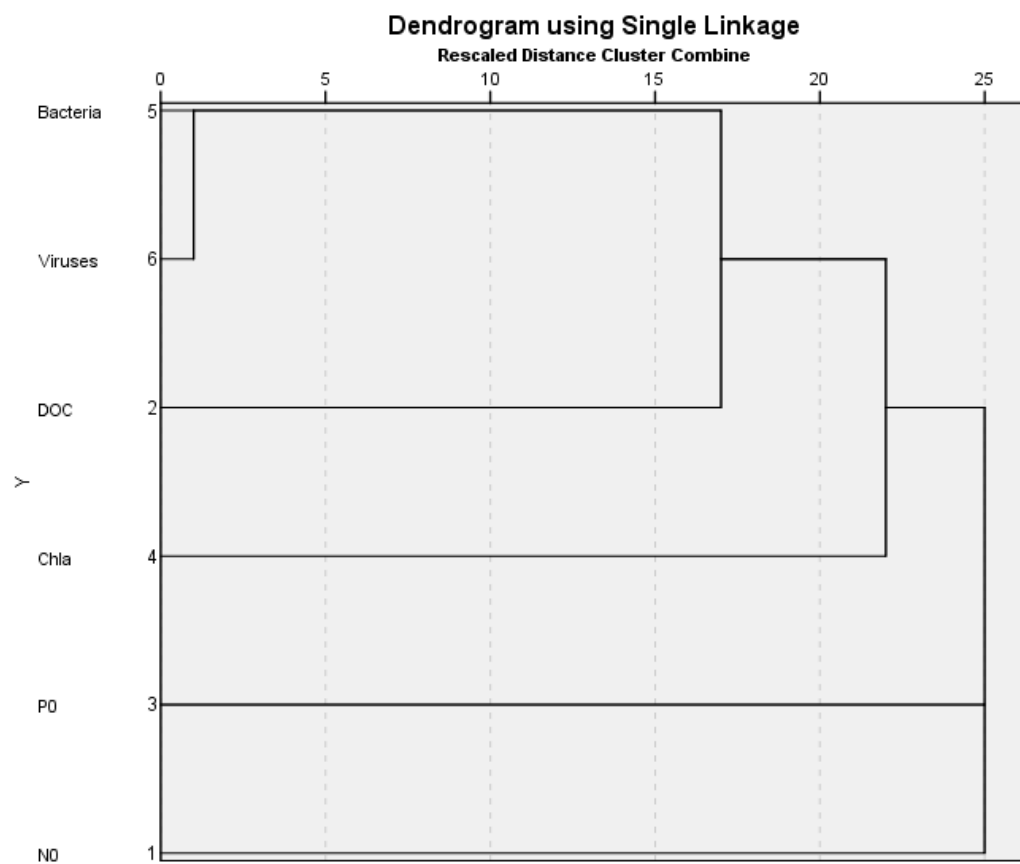

Supplement: Supplemental Information 11 — Dendrograms using single linkage of the cluster analysis for the (A) spatial (sites 1, 2, 3, 4, and 5) and (B) temporal (days 1, 2, 3, and 4) data collected in the Great Barrier Reef (Australia) displaying the classification of the variables. Please note that NO and PO regards NO3−/NO2−, and HPO42−) respectively. Please note that the TDN data was not included in the temporal analysis as there was no data for days 2 and 3. [file peerj-08-10049-s011.pdf]
